# Supplementary material for: Early fibrotic niches establish tumour-permissive microenvironments
Source: Nature. 2026 Apr 22;653(8113):254–64. doi: 10.1038/s41586-026-10399-6 (PMC13149335; doi:10.1038/s41586-026-10399-6)
Supplement: Supplementary file 1 — Supplementary Information [file 41586_2026_10399_MOESM1_ESM.pdf]

nature portfolio

https://doi.org/10.1038/s41586-026-10399-6

Supplementary information

Early fibrotic niches establish tumour-permissive microenvironments

In the format provided by the authors and unedited

SUPPLEMENTARY INFORMATION

Supplementary Table 1. List of antibodies used in this study.

Supplementary Table 1. List of antibodies used in this study.

**FACS antibodies**				
**Antibody name**	**Conjugation**	**Dilution**	**Supplier**	**Catalog #**
Anti-mouse CD326 (EpCAM)	PE-Cy7	1:200	Biolegend	118216
Anti-mouse CD45	APC	1:200	BD Biosciences	559864
Anti-mouse CD31	APC	1:200	BD Biosciences	551262
Anti-mouse CD31	APC-Cy7	1:200	Biolegend	102533
Anti-mouse SiglecF	BV421	1:200	Biolegend	155509
Anti-mouse SiglecF	PE	1:200	Biolegend	155505
Anti-mouse CD64	PE	1:200	Biolegend	139303
Anti-mouse MHC-II	FITC	1:200	eBioscience	11-5321-81
Anti-mouse CD11b	Biotin	1:200	Biolegend	101203
Anti-mouse Ly6G	PE	1:200	Biolegend	101207
Anti-mouse Gl3	PE	1:200	Biolegend	118107
Anti-mouse CD204	APC	1:200	Biolegend	154711
Anti-human HTII-280 IgM		1:200	Terrace Biotech	TB-27AHT2-280
Anti-human CD45	APC	1:200	Biolegend	368512
Anti-human EpCAM	FITC	1:200	Biolegend	324204
**Primary antibodies for immunofluorescent staining**				
**Antibody name**	**Specie**	**Dilution**	**Supplier**	**Catalog #**
Anti-Ager	Rat	1:200	R&D Systems	MAB1179

Anti-Itga2	Rabbit	1:200	Abcam	ab181548
Anti-cytokeratin 8 (Krt8)	Rat	1:200	DSHB	TROMA-I
Anti-prosurfactant protein C	Rabbit	1:300	Millipore	AB3786
Anti-Sox9 (EPR14335)	Rabbit	1:300	Abcam	Ab185230
Anti-α smooth muscle actin	Mouse	1:300	Sigma	A5228
Anti-α smooth muscle actin	Mouse	1:300	R&D Systems	MAB1420
Anti-Runx1/AML1 (D4A6)	Rabbit	1:100	Cell Signaling	8529
Anti-Pdgfrβ (28E1)	Rabbit	1:300	Cell Signaling	3169
Anti-F4/80	Rat	1:300	Bio-rad	MCA497GA
Anti-Ly6g	Rat	1:200	Biolegend	127601/127605
Anti-Gl3	Rat	1:200	Biolegend	118101
Anti-Pdgfrα (D1E1E)	Rabbit	1:300	Cell Signaling	3174
Anti-Lipocalin-2/NGAL	Goat	1:200	R&D Systems	AF1857
Anti-Amphiregulin	Goat	1:300	R&D Systems	AF989
Anti-Amphiregulin	Rabbit	1:200	Proteintech	16036-1-AP
Anti-CTHRC1	Rabbit	1:200	MaineHeatlh	Vli55
Anti-Ki67	Rat	1:300	Thermo Fisher	14-5698-82
Anti-Tenascin C	Rat	1:300	R&D Systems	MAB2138-SP
Anti-MSR1 (E4H1C)	Rabbit	1:600	Cell Signaling	91119T
Anti-CD68/SR-D1	Rabbit	1:300	R&D Systems	MAB101141-SP
Anti-LpCat1	Rabbit	1:200	Proteintech	16112-1-AP

Anti-CD177	Rabbit	1:200	R&D Systems	MAB8186
Anti-CD11c	Hamster	1:200	BioLegend	117301
Anti-Foxp3	Rabbit	1:200	Cell Signaling	12653
Anti-CD64	Rat	1:200	BioLegend	161002
Anti-Slc7a11	Rabbit	1:200	ThermoFisher	PA1-16893
Anti-MHCII	Rat	1:200	ThermoFisher	16-5321-81
Anti-PD-L1	Rabbit	1:200	R&D Systems	MAB90781-100

Secondary antibodies for immunofluorescent staining

**Antibody name**	**Dilution**	**Supplier**	**Catalog #**
Donkey anti-rat Alexa Fluor™ 647	1:500	Invitrogen	A48272
Donkey anti-rabbit Alexa Fluor™ 647	1:500	Invitrogen	A31573
Donkey anti-goat Alexa Fluor™ 647	1:500	Invitrogen	A21447
Donkey anti-mouse Alexa Fluor™ 647	1:500	Invitrogen	A-31571
Donkey anti-rabbit DyLight™ 755	1:200	Invitrogen	SA5-10043
Donkey anti-rat DyLight™755	1:200	Invitrogen	SA5-10031
Donkey anti-Rabbit Alexa Fluor™ Plus 405	1:200	Invitrogen	A48258
Donkey anti-Goat Alexa Fluor™ Plus 405	1:200	Invitrogen	A48259
Goat anti-Armenian Hamster Alexa Fluor® 647	1:500	Jackson ImmunoResearch	127-605-099
