# Supplementary material for: Early fibrotic niches establish tumour-permissive microenvironments
Source: Nature. 2026 Apr 22;653(8113):254–64. doi: 10.1038/s41586-026-10399-6 (PMC13149335; doi:10.1038/s41586-026-10399-6)
Supplement: Supplementary file 2 — Reporting Summary [file 41586_2026_10399_MOESM2_ESM.pdf]

nature portfolio

Corresponding author(s): Joo-Hyeon Lee

Last updated by author(s): Feb 06, 2026

Reporting Summary

Nature Portfolio wishes to improve the reproducibility of the work that we publish. This form provides structure for consistency and transparency in reporting. For further information on Nature Portfolio policies, see our Editorial Policies and the Editorial Policy Checklist.

Statistics

For all statistical analyses, confirm that the following items are present in the figure legend, table legend, main text, or Methods section.

n/a	Confirmed	
☐	☒	The exact sample size (*n*) for each experimental group/condition, given as a discrete number and unit of measurement
☐	☒	A statement on whether measurements were taken from distinct samples or whether the same sample was measured repeatedly
☐	☒	The statistical test(s) used AND whether they are one- or two-sided<br>*Only common tests should be described solely by name; describe more complex techniques in the Methods section.*
☒	☐	A description of all covariates tested
☐	☒	A description of any assumptions or corrections, such as tests of normality and adjustment for multiple comparisons
☐	☒	A full description of the statistical parameters including central tendency (e.g. means) or other basic estimates (e.g. regression coefficient) AND variation (e.g. standard deviation) or associated estimates of uncertainty (e.g. confidence intervals)
☐	☒	For null hypothesis testing, the test statistic (e.g. *F*, *t*, *r*) with confidence intervals, effect sizes, degrees of freedom and *P* value noted<br>*Give P values as exact values whenever suitable.*
☒	☐	For Bayesian analysis, information on the choice of priors and Markov chain Monte Carlo settings
☒	☐	For hierarchical and complex designs, identification of the appropriate level for tests and full reporting of outcomes
☒	☐	Estimates of effect sizes (e.g. Cohen's *d*, Pearson's *r*), indicating how they were calculated

Our web collection on statistics for biologists contains articles on many of the points above.

Software and code

Policy information about availability of computer code

Data collection	Leica Application Suite X (Confocal microscopy; version 2.0.0)<br>BD FACS Diva (Flow Cytometry 8.0.1)<br>FASTQ files of scRNA-seq data generated on the 10X Chromium platform were processed using the standard Cell Ranger pipeline.
Data analysis	Fiji/ImageJ (for image processing and analysis; 1.54p)<br>FlowJo (for Flow cytometry analysis; FlowJo v10.8.1)<br>GraphPad Prism (v.10.4.0 and v7.0)<br>R studio (for scRNA-seq analysis; version 4.4.2)<br>CellRanger (v.6.0.2; v7.2.0 and v8.0.0)<br>Scanpy pipeline (v.1.9.1)<br>Seurat package (v.5)

For manuscripts utilizing custom algorithms or software that are central to the research but not yet described in published literature, software must be made available to editors and reviewers. We strongly encourage code deposition in a community repository (e.g. GitHub). See the Nature Portfolio guidelines for submitting code & software for further information.

Data

Policy information about availability of data

All manuscripts must include a data availability statement. This statement should provide the following information, where applicable:

- Accession codes, unique identifiers, or web links for publicly available datasets
- A description of any restrictions on data availability
- For clinical datasets or third party data, please ensure that the statement adheres to our policy

Single-cell RNA sequencing datasets have been deposited in the Gene Expression Omnibus (GEO) under the following accession numbers: human alveolar organoids (GSE310335); mesenchymal (GSE316241) and immune (GSE316243) cells from Confetti and Red2Kras lungs; and tumours and stromal cells from Aregflox/+ and Aregflox/flox lungs (GSE316244). Additionally, publicly available datasets were used in this study and re-analyzed: Bleomycin injury scRNA-seq dataset (GSE132771); Human LUAD scRNA-seq dataset (GSE131907)

Research involving human participants, their data, or biological material

Policy information about studies with human participants or human data. See also policy information about sex, gender (identity/presentation), and sexual orientation and race, ethnicity and racism.

Reporting on sex and gender	All human specimens were obtained in de-identified form, and no donor sex or gender information was provided
Reporting on race, ethnicity, or other socially relevant groupings	No race, ethnicity or other socially constructed or socially relevant categorization was used in this manuscript.
Population characteristics	Samples included non-diseased background lung tissue and early-stage lung adenocarcinoma specimens as specified in the relevant figure legends.
Recruitment	No participants were recruited for this study.
Ethics oversight	All human specimens were obtained and approved under Research Tissue Bank Generic REC approval (Tissue Bank Project number T02233), MSKCC Institutional Review Board approval (IRB # 12-245), and Severance Hospital Review Board approval (IRB # 4-2019-0447, 4-2012-0685 and 4-2013-0770).

Note that full information on the approval of the study protocol must also be provided in the manuscript.

Field-specific reporting

Please select the one below that is the best fit for your research. If you are not sure, read the appropriate sections before making your selection.

☒ Life sciences ☐ Behavioural & social sciences ☐ Ecological, evolutionary & environmental sciences

For a reference copy of the document with all sections, see nature.com/documents/nr-reporting-summary-flat.pdf

Life sciences study design

All studies must disclose on these points even when the disclosure is negative.

Sample size	Sample size for animal and organoid experiments was made as large as possible and sufficient to determine statistical significance. For most experiments, a minimum of N=3 of biological replicates was used (except when stated otherwise in the method section of respective figure legends).
Data exclusions	No animals were excluded from the statistical analysis
Replication	For each experiment, several replicates were used (and stated in the respective figure legends).
Randomization	For all experiments both female and male mice were used. Mice were randomly assigned to experimental and control groups when possible For in vitro experiments, individual wells were randomly assigned as control or treated for all experiments.
Blinding	Blinding of both animal and organoid experiments was challeging since the same investigator was responsible for performing the experiment, collecting and processing the samples and analyze the data. To decrease unwanted biases whenever possible, mice samples were analyzed before confirming their experimental group or genotype. However, blinding was not performed when treatment effects on tumour volume were readily distinguishable between groups.

Reporting for specific materials, systems and methods

We require information from authors about some types of materials, experimental systems and methods used in many studies. Here, indicate whether each material, system or method listed is relevant to your study. If you are not sure if a list item applies to your research, read the appropriate section before selecting a response.

Materials & experimental systems

n/a	Involved in the study
☐	☒ Antibodies
☐	☒ Eukaryotic cell lines
☒	☐ Palaeontology and archaeology
☐	☒ Animals and other organisms
☒	☐ Clinical data
☒	☐ Dual use research of concern
☒	☐ Plants

Methods

n/a	Involved in the study
☒	☐ ChIP-seq
☐	☒ Flow cytometry
☒	☐ MRI-based neuroimaging

Antibodies

Antibodies used

For FACS:
Anti-mouse CD326 (EpCAM); PE-Cy7; (Biolegend #118216); clone G.8.8
Anti-mouse CD45; APC; (BD Biosciences #559864); clone 30-F11
Anti-mouse CD31; APC; (BD Biosciences #551262); clone MEC 13.3
Anti-mouse CD31; APC-Cy7; (Biolegend #102533); clone MEC 13.3
Anti-mouse SiglecF; BV421; (Biolegend #155509); clone S17007L
Anti-mouse SiglecF; PE; (Biolegend #155505); clone S17007L
Anti-mouse CD64; PE; (Biolegend #139303); clone x54-5/7.1
Anti-mouse MHC-II; FITC; (eBioscience #11-5321-81); clone M5/114.15.2
Anti-mouse CD11b; Biotin; (Biolegend #101203); clone M1/70
Anti-mouse Ly6G; PE; (Biolegend #101207); clone 1A8
Anti-mouse Gl3; PE; (Biolegend #118107); clone LG.3A10
Anti-mouse CD204; APC; (Biolegend #154711); clone 1F8C33
Anti-human HTII-280 IgM; (Terrace Biotech # TB-27AHT2-280);
Anti-human CD45; APC; (Biolegend #368512); clone 2D1
Anti-human EpCAM; FITC; (Biolegend #324204); clone 9C4

For IF staining in mouse and Human tissue samples (primary antibodies):
Anti-Ager; Rat; (R&D Systems #MAB1179); clone 175410
Anti-Itga2; Rabbit; (Abcam #ab181548); clone EPR17338
Anti-cytokeratin 8 (Krt8); Rat; (DSHB #TROMA-I)
Anti-prosurfactant protein C; Rabbit; (Millipore #AB3786)
Anti-Sox9; Rabbit; (Abcam #Ab185230); clone EPR14335
Anti-α smooth muscle actin; Mouse; (Sigma #A5228); clone 1A4
Anti-α smooth muscle actin; Mouse; (R&D Systems #MAB1420); clone 1A4
Anti-Runx1/AML1; Rabbit; (Cell Signaling #8529); clone D4A6
Anti-Pdgfrβ; Rabbit; (Cell Signaling #3169); clone 28E1
Anti-F4/80; Rat; (Bio-rad #MCA497GA); clone A3-1
Anti-Ly6g; Rat; (Biolegend #127601/127605); clone 1A8
Anti-Gl3; Rat; (Biolegend #118101); clone GL3
Anti-Pdgfrα; Rabbit; (Cell Signaling #3174); clone D1E1E
Anti-Lipocalin-2/NGAL; Goat; (R&D Systems #AF1857)
Anti-Amphiregulin; Goat; (R&D Systems #AF989)
Anti-Amphiregulin; Rabbit; (Proteintech #16036-1-AP)
Anti-CTHRC1; Rabbit; (MaineHeatlh #Vli55); clone Vli55
Anti-Ki67; Rat; (Thermo Fisher #14-5698-82); clone S01A15
Anti-Tenascin C; Rat; (R&D Systems #MAB2138-SP); clone 578
Anti-MSR1; Rabbit; (Cell Signaling #91119T); clone E4H1C
Anti-CD68/SR-D1; Rabbit; (R&D Systems #MAB101141-SP); clone 2449D
Anti-LpCat1; Rabbit; (Proteintech #16112-1-AP);
Anti-CD177; Rabbit; (R&D Systems #MAB8186); clone 1171A
Anti-CD11c; Hamster; (BioLegend #117301); clone N418
Anti-Foxp3; Rabbit; (Cell Signaling #12653); clone D608R
Anti-CD64; Rat ; (BioLegend #161002); clone S18017D
Anti-Slc7a11; Rabbit; (ThermoFisher #PA1-16893); clone PA1-16893
Anti-MHCII; Rat; (ThermoFisher #16-5321-81); clone M5/114.15.2
Anti-PD-L1; Rabbit; (R&D Systems #MAB90781-100); clone 2096C

For IF staining in mouse and Human tissue samples (secondary antibodies):
Donkey anti-rat Alexa Fluor™ 647; (Invitrogen #A48272)
Donkey anti-rabbit Alexa Fluor™ 647; (Invitrogen #A31573)
Donkey anti-goat Alexa Fluor™ 647; (Invitrogen #A21447)
Donkey anti-mouse Alexa Fluor™ 647; (Invitrogen #A-31571)
Donkey anti-rabbit DyLight™ 755; (Invitrogen #SA5-10043)
Donkey anti-rat DyLight™755; (Invitrogen #SA5-10031)

Donkey anti-Rabbit Alexa Fluor™ Plus 405; (Invitrogen #A48258)
Donkey anti-Goat Alexa Fluor™ Plus 405; (Invitrogen #A48259)
Goat anti-Armenian Hamster Alexa Fluor® 647; (Jackson ImmunoResearch #127-605-099)

Validation

All antibodies were tested and validated by the manufactor (details are specified in supplier webpages and are accessible using catalog numbers indicated for each antibody above). Antibodies which were not previously validated in mouse or Human tissue were tested for this study and data presented in the submitted manuscript.

Anti-Ager: validated for Immunohistochemistry, Western Blot. Tested reactivity: Mouse, Rat
Anti-Itga2: validated for use in Western Blot, Flow Cytometry, Flow Cytometry, Immunoprecipitation, Immunohistochemistry, Immunocytochemistry/immunofluorescence. KO validated for confirmed specificity. Tested reactivity: Human, Mouse, Rat.
Anti-Krt8: specificity for keratin K8 has been knock-out validated. Tested reactivity: Canine, Human, Mouse
Anti-Sox9: validated for use in Flow Cyt (Intra), ICC/IF, IHC-P, IP, WB. Tested reactivity: Human, mouse, rat samples.
Anti-a-smooth actin: Multiplex Immunofluorescence, Immunohistochemistry, Western Blot, Intracellular Staining by Flow Cytometry, Dual RNAscope ISH-IHC Compatible, Immunocytochemistry, Simple Western, CyTOF-ready; Tested reactivity: Human, Mouse, Rat
Anti-Runx1: Western Blotting. Tested reactivity: Human, Mouse.
Anti-Pdgfrβ: Western Blotting, Simple Western™, Immunoprecipitation, Immunohistochemistry (Paraffin), Immunofluorescence (Frozen), Immunofluorescence (Immunocytochemistry). Tested reactivity: Human, Mouse, Rat. This antibody may cross-react with PDGF receptor α when highly overexpressed. Nonspecific labeling in fixed frozen mouse colon has been observed by immunofluorescence.
Anti-F4/80: This product has been reported to work in the following applications. This information is derived from testing within our laboratories, peer-reviewed publications or personal communications from the originators: Flow Cytometry, Immuno-electron Microscopy, Immunofluorescence, Immunohistology, Western Blotting and Radioimmunoassays. Tested reactivity: Mouse.
Anti-Ly6g: Each lot of this antibody is quality control tested by immunofluorescent staining with flow cytometric analysis. Tested reactivity: Mouse.
Anti-Gl3: The GL3 antibody has been shown to be useful in identifying γ/δ T cells by flow cytometry and immunohistochemistry and depleting γ/δ T cells in vivo. Tested reactivity: Mouse.
Anti-Pdgfrα: Western Blotting, Immunoprecipitation, Immunohistochemistry (Paraffin), Immunofluorescence (Immunocytochemistry), Flow Cytometry (Fixed/Permeabilized). Tested reactivity: Human, Mouse.
Anti-Lipocalin-2/NGAL: Immunohistochemistry, Western Blot, Simple Western, Immunoprecipitation; Tested reactivity: Mouse.
Anti-Amphiregulin (mouse): Immunohistochemistry, Western Blot, ELISA Capture (Matched Antibody Pair), Neutralization. Tested reactivity: Mouse, Guinea Pig, Transgenic Mouse
Anti-Amphiregulin (Human): KD/KO validated. WB, IHC, IF/ICC, IF-P, FC (Intra), IP, ELISA. Tested reactivity: human, mouse.
Anti-Cthrc1: The antibody was raised against the whole molecule of human Cthrc1. It cross-reacts with rat and mouse Cthrc1. Western-blot, ELISA, IP.
Anti-Ki67: Immunohistochemistry (Paraffin) (IHC (P)), Immunohistochemistry (Frozen) (IHC (F)), Immunocytochemistry (ICC/IF). Dog, Cynomolgus. Tested reactivity: monkey, Human, Mouse, Non-human primate, Rat.
Anti-Tenascin C: Western Blot, Neutralization, Immunocytochemistry. Tested reactivity: Human, Mouse.
Anti-MSR1: Immunofluorescence (Frozen), Immunofluorescence (Immunocytochemistry), Flow Cytometry (Fixed/Permeabilized) and Flow Cytometry (Live). Tested reactivity: Mouse.
Anti-CD68: Multiplex Immunofluorescence, Immunohistochemistry, Intracellular Staining by Flow Cytometry, CyTOF-ready. Tested reactivity: Human, Mouse, Transgenic Mouse.
Anti-LpCat1: KD/KO validated. WB, IHC, IF/ICC, IP, CoIP, ELISA. Tested reactivity: human, mouse, rat.
Anti-CD177: Flow Cytometry; Tested reactivity: mouse.
Anti-Cd11c: Tested reactivity: Mouse; Each lot of this antibody is quality control tested by immunofluorescent staining with flow cytometric analysis.
Anti-Foxp3: IHC Leica Bond, Immunohistochemistry (Paraffin), Immunofluorescence (Frozen) and Flow Cytometry (Fixed/Permeabilized). Tested reactivity: Mouse, Monkey.
Anti-CD64: FC - Quality tested, IHC-F – Verified. Tested reactivity: Mouse and Human.
Anti-Slc7a11: Western Blot (WB), Immunohistochemistry (Paraffin) (IHC (P)), Immunocytochemistry (ICC/IF), Flow Cytometry (Flow), In Situ Hybridization (ISH). Tested reactivity: Human, Mouse, Rat
Anti-MHCII: Flow Cytometry (Flow), Neutralization (Neu) and Functional Assay (Functional). Tested reactivity: Mouse.
Anti-PD-L1: Immunohistochemistry, Western Blot, Flow Cytometry; Tested reactivity: Mouse

Eukaryotic cell lines

Policy information about cell lines and Sex and Gender in Research

Cell line source(s)

Primary mouse lung cells were derived from wildtype or transgenic mouse lungs in the laboratory. Primary human lung cells were derived from fresh human lung tissues in the laboratory. HEK293T cells were obtained from ATCC (CRL-11268) and maintained according to the supplier's recommendations.

Authentication

All cell lines generated in-house were genotyped for authentication. HEK293T cells were not additionally authenticated.

Mycoplasma contamination

All cell lines used tested negative for Mycoplasma contamination.

Commonly misidentified lines
(See ICLAC register)

No.

Animals and other research organisms

Policy information about studies involving animals; ARRIVE guidelines recommended for reporting animal research, and Sex and Gender in Research

Laboratory animals	Sftpc-CreERT2 (Jax: 028054), R26R-Confetti (Jax: 013731), Pdgfra-CreERT2 (Jax: 032770), R26R-iDTR (Jax: 007900), NOD/Scid Il2rg null Tg (NSGj Jax: 005557), and Ai6/RCL-ZsGreen (Jax: 007906) animals were obtained from The Jackson Laboratory. AregloxP/loxP animals were kindly provided by Prof. Menna Clatworthy from the University of Cambridge, UK. Red2Kras mice were generated inhouse and previously described. CCR2-CreERT2 mice were kindly provided by Prof. Burkhard Becher (University of Zurich). All transgenic mouse strains were maintained on a C57BL or C57BL/6Brd-Tyr 597 c-Brd mixed background. Mice were housed under specific pathogen-free conditions in individually ventilated cages with a 12-hour light/12-hour dark cycle. Ambient temperature was maintained at 20–24 °C with relative humidity of 40–60%, in accordance with institutional animal care guidelines.
Wild animals	No wild animals were used in this study
Reporting on sex	Both male and female animals were used for all experiments perfomed.
Field-collected samples	No filed collected samples
Ethics oversight	Mouse studies in the UK were approved under UK Home Office Project Licences PC7F8AE82 and PP3176550, and experiments in the US and Korea were approved by the MSKCC Institutional Animal Care and Use Committee (IACUC) (protocol #24-04-003) and GIST IACUC (protocol # GIST-2022-043). All procedures complied with institutional and national guidelines.

Note that full information on the approval of the study protocol must also be provided in the manuscript.

Plants

Seed stocks	n/a
Novel plant genotypes	n/a
Authentication	n/a

Flow Cytometry

Plots

Confirm that:

☒ The axis labels state the marker and fluorochrome used (e.g. CD4-FITC).

☒ The axis scales are clearly visible. Include numbers along axes only for bottom left plot of group (a 'group' is an analysis of identical markers).

☒ All plots are contour plots with outliers or pseudocolor plots.

☒ A numerical value for number of cells or percentage (with statistics) is provided.

Methodology

Sample preparation	For isolation of lung cell, mice were culled by cervical dislocation and lungs cleared of blood via perfusion with 10ml of PBS. Lungs were inflated with 2-3ml of a Dispase solution (Fisher Scientific, 11553550) via intratracheal injection. When isolating mesenchymal cells, Collagenase I (GIBCO, 17100017) was added to the Dispase solution at 350U/ml before inflation. Lungs were carefully dissected out of the thoracic cavity and placed on a petri dish on ice. Individual lobes were separated from each lung, placed in a 50ml falcon and minced into small pieces. Cells were washes down with 3ml of PBS to the bottom of the falcon tube. When isolating epithelial cells only, 60ul of 100mg/ml Collagenase/Dispase solution was added per tube. Samples were places in a shaking incubator at 37ºC, 190 rpm, for 45 min. 7.5ul of 1% DNase I (Sigma, D4527) was added to each sample in the final 10 min of incubation. The resulting cell suspensions were filtered sequentially through 100 µm and 40 µm cell strainers and washed with 2 ml of 10% foetal bovine serum (FBS, 815 Pan-Biotech, P40- 37500) in PBS (PF10) to collect remaining cells. Samples were centrifuged at 800 rpm for 5 min at 4ºC. Supernatant was removed and pellets were resuspended in 1 ml of red blood cell lysis buffer (RBC buffer, made inhouse: 150 mM NH_4Cl and 10 mM 567 $KHCO_3$ in distilled H_2O) for 60 seconds at RT. After lysis, 6 ml of Dulbecco's Modified Eagle Medium Nutrient Mixture F-12 820 (DMEM/F-12, Invitrogen, 11330057) was added to the tube to neutralize the RBC buffer. 500µL of filtered FBS was added slowly to the bottom of each tube to collect live cells. Tubes were centrifuged again at 800rpm for 5 min at 4ºC. Cell pellets were resuspended in PF10 and placed into separate 1.5 ml tubes for antibody staining.

Instrument	BD Influx™ cell sorter<br>Facsdiscover s8<br>Facsymphony s6
Software	Raw FACS files were obtained from sorter and analysed using FlowJo
Cell population abundance	In the lung tissue, EPCAM+ cells comprise approximately 15% of the single cell suspension. For Red2Kras mice, RFP+ cells comprise approximately 10-30% of the epithelial populations (which is dependent on the time point used for collection, dose of tamoxifen, and mouse to mouse variability).
Gating strategy	The gating strategy for each experiment and population of interest is detailed in the method sections.<br>To isolate RFP+ mutant cells from Red2Kras animals the following strategy was used: Epcam+/CD45-/CD31- -> RFP+/YFP-.<br>To isolate lineage labelled fibroblasts the following strategy was used: Epcam-/CD45-/CD31- -> ZsGreen+<br>To isolate mesenchymal cells the following strategy was used: Epcam-/CD45-/CD31-<br>To isolate alveolar macrophages the following strategy was used: CD64+/CD45+ -> SiglecF+<br><br>Gating strategies used to isolate cells for scRNA-seq analyses are provided in the Supplementary Information

☒ Tick this box to confirm that a figure exemplifying the gating strategy is provided in the Supplementary Information.
